# Flexible, Highly Thermally Conductive and Electrically Insulating Phase Change Materials for Advanced Thermal Management of 5G Base Stations and Thermoelectric Generators

**DOI:** 10.1007/s40820-022-01003-3

**Published:** 2023-01-09

**Authors:** Ying Lin, Qi Kang, Yijie Liu, Yingke Zhu, Pingkai Jiang, Yiu-Wing Mai, Xingyi Huang

**Affiliations:** 1 Shanghai Key Laboratory of Electrical Insulation and Thermal Ageing, Department of Polymer Science and Engineering, the State Key Laboratory of Metal Matrix Composites, Shanghai Jiao Tong University, Shanghai, 200240 People’s Republic of China; 2 Centre for Advanced Materials Technology (CAMT), School of Aerospace, Mechanical and Mechatronic Engineering J07, The University of Sydney, Sydney, NSW 2006 Australia

**Keywords:** Coaxial electrospinning, Boron nitride nanosheets, Phase change nanocomposites, Thermal conductivity, Thermal management

## Abstract

Thermal management has become a crucial problem for high-power-density equipment and devices. Phase change materials (PCMs) have great prospects in thermal management applications because of their large capacity of heat storage and isothermal behavior during phase transition. However, low intrinsic thermal conductivity, ease of leakage, and lack of flexibility severely limit their applications. Solving one of these problems often comes at the expense of other performance of the PCMs. In this work, we report core–sheath structured phase change nanocomposites (PCNs) with an aligned and interconnected boron nitride nanosheet network by combining coaxial electrospinning, electrostatic spraying, and hot-pressing. The advanced PCN films exhibit an ultrahigh thermal conductivity of 28.3 W m^−1^ K^−1^ at a low BNNS loading (i.e., 32 wt%), which thereby endows the PCNs with high enthalpy (> 101 J g^−1^), outstanding ductility (> 40%) and improved fire retardancy. Therefore, our core–sheath strategies successfully balance the trade-off between thermal conductivity, flexibility, and phase change enthalpy of PCMs. Further, the PCNs provide powerful cooling solutions on 5G base station chips and thermoelectric generators, displaying promising thermal management applications on high-power-density equipment and thermoelectric conversion devices.

**Supplementary Information:**

The online version contains supplementary material available at 10.1007/s40820-022-01003-3.

## Highlights

- A core–sheath structured phase change nanocomposite (PCN) with aligned and overlapping interconnected BNNS networks were successfully fabricated.
- The PCN has an ultrahigh in-plane thermal conductivity (28.3 W m^−1^ K^−1^), excellent flexibility and high phase change enthalpy (101 J g^−1^).
- The PCN exhibits intensively potential applications in the thermal management of 5G base stations and thermoelectric generators.

**Supplementary Information:**

The online version contains supplementary material available at 10.1007/s40820-022-01003-3.

## Graphical abstract

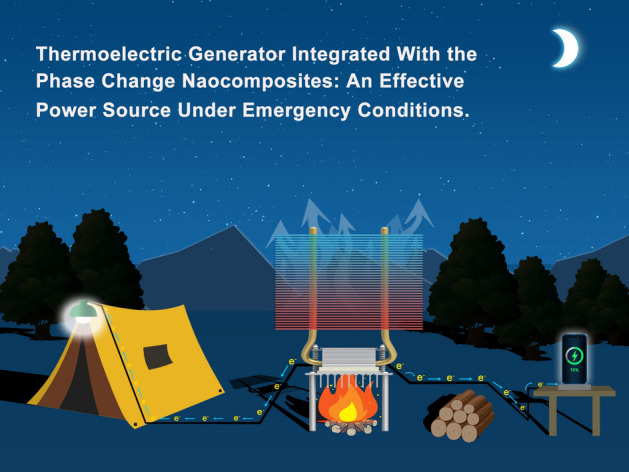

## Introduction

Heat dissipation becomes a great challenge for power equipment and electronic devices with their continuous evolution toward miniaturization, high integration and increasing power density [1–3]. Over-heating caused by heat accumulation can significantly reduce the operating efficiency, reliability and life span of these equipment and devices, and even cause fire risks [4–8]. Today, numerous engineering fields, including power generation/delivery, energy storage/conversion, integrated circuits and aircrafts, have ever-increasing demands for high performance thermal management materials [9–16]. In this context, the high-thermal-conductivity materials with excellent mechanical, electrical insulation and fire-retardant performance are highly desirable to resolve the thermal issues.

Phase change materials (PCMs), as ‘passive’ thermal management materials, have been applied extensively in various fields due to their large heat storage capacity and isothermal behavior during phase transition [17–19]. Organic PCMs, in particular, paraffin and polyethylene glycol (PEG), have attracted much attention because of their high latent heat, excellent cycle stability and non-toxicity [20, 21]. However, these PCMs also have several critical issues including low intrinsic thermal conductivity, leakage problems and lack of flexibility, which severely limits their applications [22–24]. To overcome these problems, many approaches have been proposed. For example, highly thermally conductive fillers (e.g., graphene, carbon nanotubes) were intensively used to enhance thermal conductivity of PCMs [25–27]. Packaging strategies based on microcapsules, porous confinement and polymer skeletons have been used to solve the problem of leakage during phase change [4, 28–30].

Although significant achievements have been made in enhancing the thermal conductivity and reducing the leakage of PCMs, it is still a great challenge to simultaneously retain their flexibility and large phase transition enthalpy [6, 31]. Constructing highly interconnected heat pathways within PCMs using high-aspect-ratio nanofillers and encapsulating such PCMs with flexible polymers as supporting materials hold the keys to solving these challenging issues [32–34]. Coaxial electrospinning is a powerful way to manufacture core–sheath fibers which can encapsulate the PCMs to avoid leakage [35]. However, the existing phase change composites (PCCs) fabricated by coaxial electrospinning usually exhibit low thermal conductivity enhancement, since there is a lack of effective control over the filler loading and material morphology [23].

Electrical insulation is critical when the thermal management materials work under an applied electric field. In this case, nano-carbon and metallic filler cannot be used because of their high electrical conductivity enhancement effect on the composite materials. Hexagonal boron nitride nanosheet (*h*-BNNS) is an ideal filler because of its ultrahigh thermal conductivity, high radius-to-thickness ratio, wide band gap and low density [36, 37]. Herein, highly thermally conductive phase change nanocomposite (PCN) films with an aligned and overlapping interconnected BNNS network were prepared combining coaxial electrospinning, electrostatic spraying (designated as ‘es’) and hot-pressing in sequence. The PCNs have a core–sheath structure, where the core is PEG and the sheath consists of BNNS-based thermoplastic polyurethane (TPU) nanocomposites. The TPU-containing sheath layer supports and encapsulates the phase change components, and endows the nanocomposites with excellent flexibility. The BNNSs are highly interconnected and aligned in the in-plane direction of the fibrous non-woven fabric, facilitating the resultant PCN films with an ultrahigh in-plane thermal conductivity of 28.3 W m^−1^ K^−1^ at a low BNNS loading of 32 wt%. Furthermore, the PCN films exhibit a high latent heat of > 101 J g^−1^, good fire retardancy and electrical insulation. Finally, we demonstrate the excellent thermal management applications of the PCN films for the fifth generation (5G) of cellular technology base stations and thermoelectric generators.

## Experimental Section

### Chemicals and Materials

Boron nitride (BN) powders with an average diameter of 3 μm were obtained from 3 M Technical Ceramics (USA). Thermoplastic polyurethane (TPU) was provided by BASF Co., Ltd. (China) and polyethylene glycol (AR, *M*_w_ = 10,000) was purchased from Shanghai Titan Scientific Co. Ltd (China). *N,N*-dimethylformamide (DMF, AR), tetrahydrofuran (THF, AR), dichloro-methane and isopropanol (AR) were obtained from Sinopharm Chemical Reagent Co., Ltd. (China). All the chemical reagents were used as received. Deionized (DI) water was prepared in the laboratory.

### Fabrication of Aligned PCN Fibers: PEG@TPU/BNNS

Boron nitride nanosheets (BNNSs, average diameter of ~ 1 μm, see Fig. S1) were obtained by sonication-assisted liquid phase exfoliation of boron nitrides (BNs) in accordance with previously reported studies [36, 38]. Aligned PCN fibers with a core–sheath structure were fabricated by coaxial electrospinning. Specifically, the precursor solution as the sheath layer was prepared by dissolving TPU in a mixture solvent of *N,N*-dimethylformamide (DMF) and tetrahydrofuran (THF) (1:1) by magnetic stirring at 60 °C for 2 h; and the core precursor solution was PEG solution at a concentration of ~ 60% (w/v). Further, to fabricate the TPU/BNNS composite sheath layer, a given amount of BNNS was dispersed in a mixture solvent comprising DMF and THF with ultrasonic treatment for 1 h, and then added into the sheath layer precursor solution; the mixtures were stirred at room temperature for 12 h. In the sheath layer precursor mixture, the concentrations of TPU and BNNS were 14 and ~ 3.5 wt%, respectively.

Then, the TPU/BNNS precursor solution and PEG solution were mounted on the coaxial spinneret to produce the sheath and core structure of the composite fibers. During electrospinning, the spinneret stainless steel coaxial needle tip to the collector was set at ~ 15 cm and the voltage ~ 15 kV. The sheath solution flow was controlled at 0.5 mL h^−1^ by a syringe pump, while the core solution flow was varied from 0.1 to 0.5 mL h^−1^. The rotational speed of the cylindrical collector was kept at ~ 1500 rpm. The electrospinning was conducted at ambient temperature (25 °C) and relative humidity (RH) between 40 and 50%. The PEG@TPU/BNNS non-woven fabric was obtained after the coaxial electrospinning by peeling off the aluminum foil and drying at room temperature in vacuum for 24 h to remove the residual solvent. The PEG@TPU nanofibers were prepared using the same coaxial electrospinning process.

### Fabrication of Phase Change Nanocomposite Film: PEG@TPU/BNNS-es

BNNSs were first dispersed in a DMF and isopropanol (1:1) mixture solvent at a concentration of 20 mg mL^−1^. Then, the BNNS suspension was fed into a plastic syringe equipped with a blunt metal needle for electrostatic spraying BNNSs onto the freshly prepared PEG@TPU/BNNS nanofibers on aluminum foil. The positive electrode of the high voltage DC power supply was clamped to the metal needle tip of the syringe with a voltage of ~ 18 kV and a working distance from tip to collector of 12 cm. The BNNS suspension flow rate was kept at 1 mL h^−1^, and the electrostatic spraying time of BNNSs was 20, 40, 60 and 80 min. In this way, PEG@TPU/BNNS nanofibers covered by a BNNS layer were obtained. Then, the resultant PEG@TPU/BNNS-es porous fabric was peeled off the aluminum foil and dried at room temperature under vacuum for 24 h to remove the solvent.

The phase change composite films PEG@TPU/BNNS-es were prepared by mold-pressing the porous fabric at room temperature under 15 MPa for 30 min and then at ~ 60 °C under 10 MPa for 30 min. For comparison, the PEG@TPU and PEG@TPU/BNNS were also treated with mold-pressing under the same conditions.

### Characterization

The microstructures of BNNSs, PCN fibers and films were characterized by field emission scanning electron microscopy (FE-SEM, Nova NanoSEM 450, FEI, USA). Field emission transmission electron microscopy (FE-TEM, JEM-2100, JEOL Corporation, Japan) was used to observe the core–sheath structure of PEG@TPU and PEG@TPU/BNNS fibers at an acceleration voltage of 200 kV. The TEM samples of phase change composite fibers were obtained by coaxial electrospinning PEG@TPU, PEG@TPU/BNNS fibers onto a copper grid, respectively, and dried before measurement. The optical images of PEG@TPU/BNNS-es and 5G base station chip were taken by a digital camera (Nikon Z50). Atomic force microscopy (AFM) was used to observe the morphology of the phase change composite fibers at room temperature and 65 °C. The samples were prepared by coaxial electrospinning fibers onto the silicon chip and air-dried before measurement. Thermogravimetric analysis (TGA) of phase change composite films was characterized using a NETZSCH TG209 F3 in the range of 50–800 °C at a heating rate of 20 °C min^−1^ under air atmosphere. The thermal diffusivity (λ, mm^2^ s^−1^) was also measured with a laser flash thermal conductivity test instrument (LFA467 HyperFlash, Netzsch). The diameter and thickness of the thermal conductive specimens were 25.4 mm and ~ 20 μm (for in-plane thermal conductivity measurement), 12.7 and ~ 0.2 mm (for through-plane thermal conductivity measurement), respectively. The specimen density (*ρ*, g cm^−3^) was obtained by the water displacement method, and the specific heat (*C*_p_, J g^−1^ K^−1^) was measured with a differential scanning calorimeter (DSC, NETZSCH 200 F3, Germany). The thermal conductivity (*κ*, W/m K) was calculated by: *κ* = *λ* × *ρ* × *C*_p_. The phase transition behaviors of the samples were characterized by DSC at a heating rate of 10 °C min^−1^ in the range of 20–90 °C under N_2_ atmosphere. Frequency-dependent electrical conductivity tests were conducted on samples, both sides of which were deposited with a copper layer as electrodes, using a high-resolution dielectric analyzer (Novocontrol Alpha-N, GmbH Concept 40 Hz) in the temperature range of −20 to 60 °C and frequencies between 10^–1^ and 10^6^ Hz. The DC breakdown strength was measured using a dielectric strength tester (DH, Shanghai Lanpotronics Co., China) with a 10 mm ball-to-plate stainless electrode system. Tensile properties were determined on a single-column materials testing system (INSTRON 3343) at a rate of 5 mm min^−1^. The surface temperature of the 5G base station was recorded by infrared thermographs (FOTRIC 220, TEquipment). The thickness of the sample (PEG@TPU/BNNS-es film) served as a TIM in the 5G base station was ~ 45 μm. Finally, the sample loaded on the cold side of a thermoelectric generator was prepared by vertically folding the PCN fibers and subsequent pressing.

## Results and Discussion

### Preparation and Characterization of the PCN Films

**Fig. 1:**
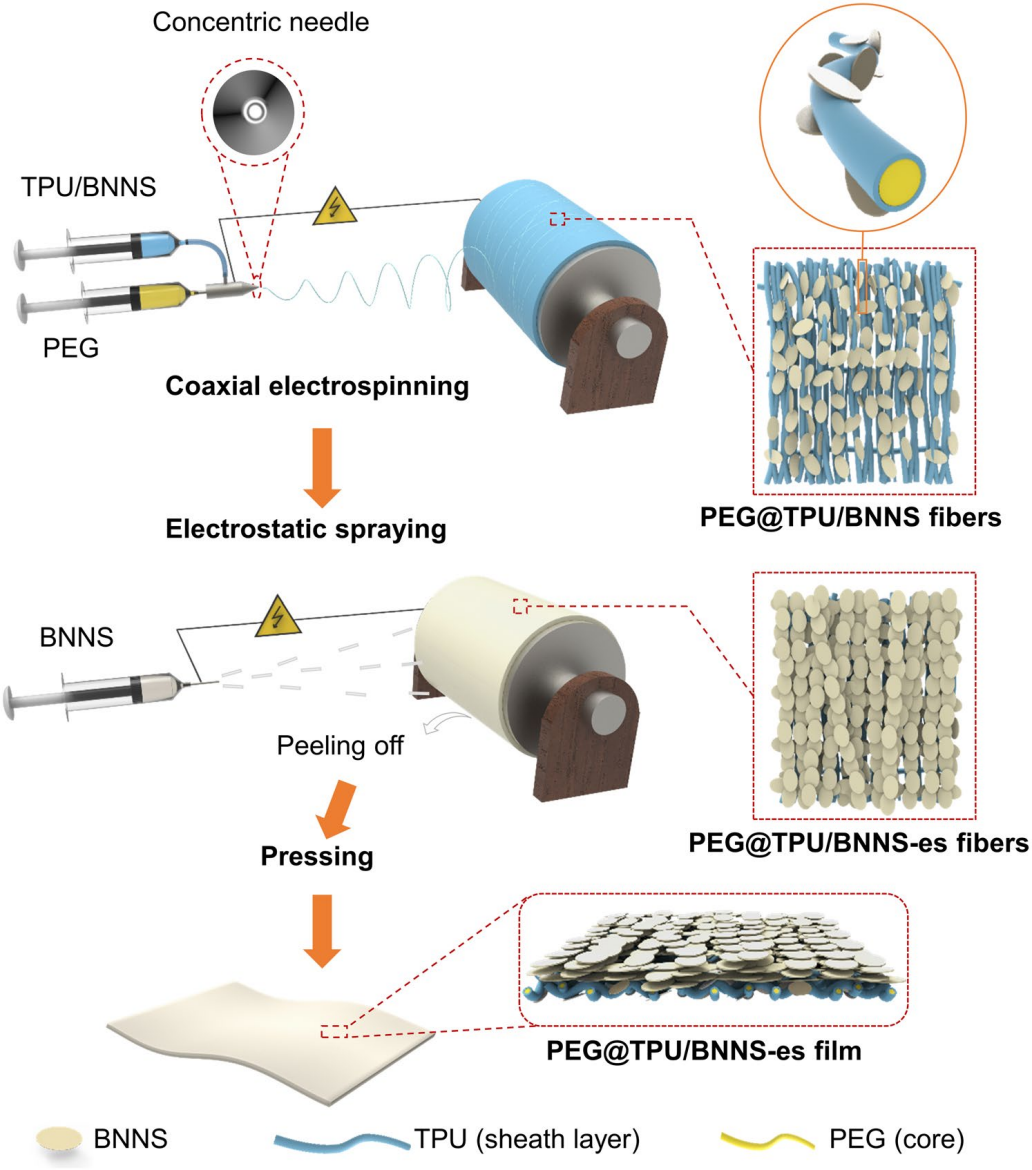
Scheme of the preparation process of the PEG@TPU/BNNS-es nanocomposite films

Coaxial electrospinning was used to construct core–sheath PEG@TPU/BNNS-es nanocomposite films (Fig. 1), aiming to endow PCMs with good flexibility and ensure their normal use above the melting point without leakage. The concentrically aligned spinneret has two separate channels for two different electrospinning solutions. The spinning solutions for the core (i.e., PEG) and the sheath layer (TPU or TPU/BNNS) were fed into the inner and outer channels of metallic capillaries, respectively. To produce the aligned core–sheath fibers, the cylindrical collector covered with aluminum foil was controlled at a high rotation speed. A suitable high voltage was applied between spinneret and collector during the coaxial spinning, which could make a stretched solution jet, ensuring uninterrupted spinning of fibers by electrostatic forces. After the electrospinning, BNNSs dispersion was sprayed onto the as-prepared PEG@TUP/BNNS non-woven fabric under a high voltage. The BNNSs tended to uniformly cover the composite fibers under electrostatic attraction. The resultant PCN (PEG@TPU/BNNS-es) films were then obtained by hot-pressing the BNNSs-covered PEG@TUP/BNNS non-woven fabric. Hot-pressing at a moderate temperature (~ 60 °C) is important to improve the properties of the PEG@TPU/BNNS-es films as it favors the densification of the PCNs.

**Fig. 2:**
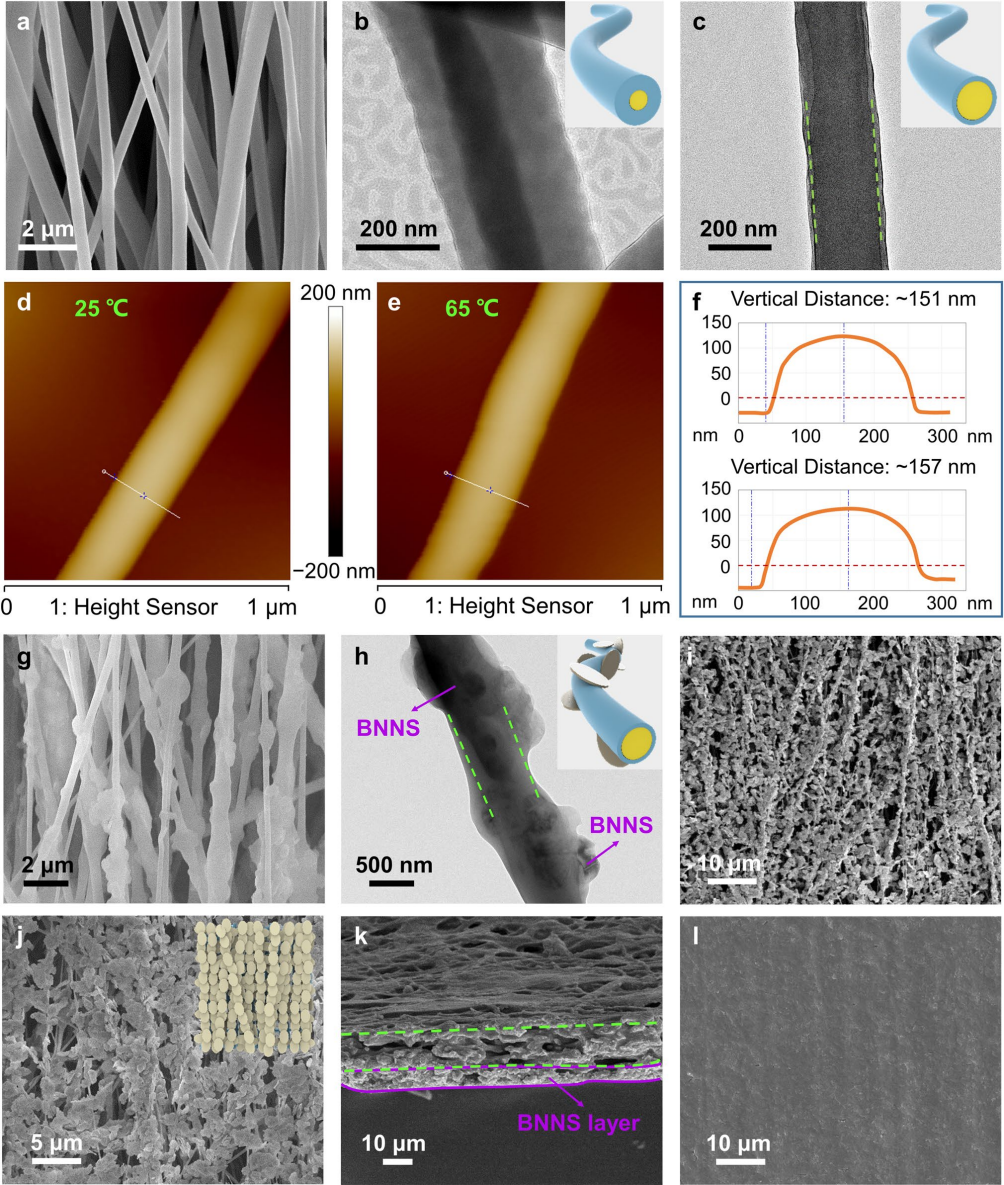
Microstructure characterization. **a** SEM image of PEG@TPU fibers. **b**, **c** TEM images of PEG@TPU fibers with different mass ratios of PEG to TPU, the insets at the top right are diagrams of the corresponding core–sheath fibers. **d, e** AFM images of the PEG@TPU fiber tested at 25 and 65 °C. **f** Corresponding AFM data of the PEG@TPU fiber at 25 (top) and 65 °C (bottom). **g** SEM image of PEG@TPU/BNNS fibers. **h** TEM image of a PEG@TPU/BNNS fiber, the inset is a diagram of the core–sheath nanocomposite fiber. **i**, **j** SEM images of PEG@TPU/BNNS-es fibrous membrane, the inset in **j** is the diagram of the corresponding fibrous membrane. **k**, **l** SEM images of the cross section and surface of the PEG@TPU/BNNS-es nanocomposite film, respectively

The PEG@TPU nanofibers have a diameter of 200–800 nm (Fig. 2a), exhibiting an aligned arrangement and typical core–sheath structures (Fig. 2b, c). The ratio of the sheath-to-core can be regulated through changing either the advancing speeds of the syringe pumps or the concentration of spinning solutions. We can see a pore in the cross section of the nanofibers after the films were soaked in water at 90 °C for 24 h (Fig. S2a, b), confirming that the PEG core has been removed in the hot water bath. This further demonstrates the core–sheath structure of the PCN fibers. Leakage was not observed when the nanofiber was heated from room temperature to 65 °C, while its diameter increased slightly due to the melting of the PEG (Fig. 2d–f). Also, the PEG@TPU/BNNS fibers show a core–sheath structure (Fig. 2g, h), and the BNNSs dispersed in the sheath layer exhibit a kebab-like morphology. In combination with the atomic force microscope (AFM) images (Fig. S2c–e), these results demonstrate that the addition of BNNSs almost does not affect the construction of the core–sheath structure in these PCN fibers. In the PEG@TPU/BNNS-es fabrics, the electrostatically sprayed BNNSs are well aligned along the nanocomposite fibers and the amount of BNNSs on the fabric surface can be controlled by the spraying time (Fig. 2i, j). As shown in Fig. S3a–d, BNNSs are isolated from each other within 60-min spraying, while a longer spraying time of 80 min can result in overlapping interconnections of the BNNSs (Fig. S3e). A much longer spraying time, however, causes a too thick BNNS layer (Fig. S3f) that is easy to separate from the nanofiber surface. After hot-pressing, the BNNSs are still oriented and continue to maintain the overlapping interconnections in the PEG@TPU/BNNS-es films (Figs. 2k, l, S4). It should be noted that the BNNS network in PEG@TPU/BNNS-es consists of two parts. One part is the BNNSs in the sheath layer, and the other part is the BNNSs due to electrospraying. Hot-pressing at a moderate temperature is essential to form the BNNS network as it cannot only densify the nanocomposite fibers, but also retain their core–sheath structure.

### Thermal Properties of the PCNs

**Fig. 3:**
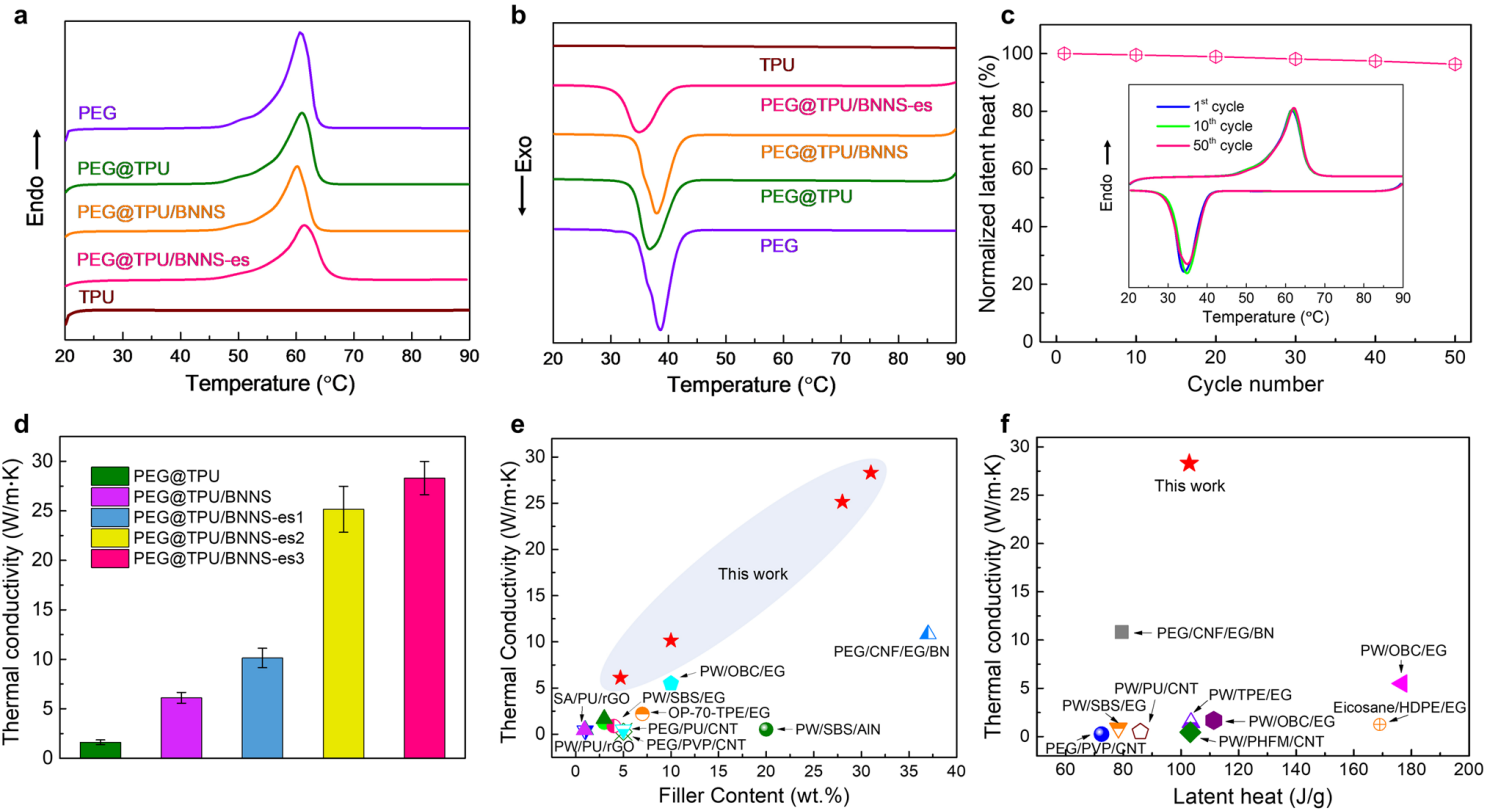
Thermal properties of various materials. DSC curves of the materials during **a** heating and **b** cooling. **c** Normalized latent heat of fusion for PEG@TPU/BNNS-es with ~ 32 wt% BNNSs, the inset shows the DSC curves for 1st, 10th and 50th thermal cycles of PEG@TPU/BNNS-es. **d** In-plane thermal conductivity of various PCCs studied in this work, PEG@TPU/BNNS-es 1–3 have a BNNS loading of about 10, 28 and 32 wt%, respectively. **e** Thermal conductivity versus filler loading of PEG@TPU/BNNS-es and reported PCCs with filler loading < 40 wt% [32, 33, 39–47]. **f** Thermal conductivity versus latent heat of PEG@TPU/BNNS-es and reported flexible PCCs [32, 33, 41–47]

Figure 3a and b presents the differential scanning calorimetry (DSC) curves of various materials from 20 to 90 °C. In heating, all materials except TPU show a melting peak of PEG at ~ 60 °C, which corresponds to the solid–liquid phase transition. Since TPU has a melting point much higher than 90 °C, no characteristic peak can be observed in this measurement temperature range. PEG@TPU exhibits a melting temperature (*T*_m_) of 61 °C and latent heat of fusion (Δ*H*_m_) of 156.5 J g^−1^, and its solidification temperature (*T*_s_) and solidification latent heat (Δ*H*_s_) are 36.8 °C and 154.1 J g^−1^ (Table S1), respectively. The enthalpy of PEG@TPU is obviously lower than that of pure PEG due to the addition of TPU as the sheath layer. With the incorporation of BNNSs, the composite nanofibers exhibit a reduced latent heat (the BNNSs content was estimated by thermogravimetric results, shown in Fig. S5). Specifically, PEG@TPU/BNNS with 4.7 wt% BNNSs can retain 84.7% and 84.6% in Δ*H*_m_ and Δ*H*_s_ of PEG@TPU, respectively. When introducing ~ 32 wt% BNNSs by electrostatic spraying, PEG@TPU/BNNS-es exhibits a Δ*H*_m_ and Δ*H*_s_ of 102.9 and 101.2 J g^−1^, respectively. Moreover, the PEG@TPU/BNNS-es show similar *T*_m_ and *T*_s_ as pure PEG, suggesting that the sheath layer only has marginal effect on the phase transition behaviors of PEG. After 50 melting–solidification cycles, the DSC curves of the PEG@TPU/BNNS-es almost coincided with the original (Fig. 3c) and the latent heat of fusion remains 96.3% of its original value, showing an excellent cyclic stability.

The PEG@TPU film shows an in-plane thermal conductivity (*κ*_//_) of about 1.62 W m^−1^ K^−1^. When incorporating 4.7 wt% BNNS into the sheath layer, the PEG@TPU/BNNS shows an enhanced *κ*_//_ of 6.11 W m^−1^ K^−1^ (Fig. 3d). Moreover, additional incorporation of BNNS layers by electrostatic spraying further enhanced *κ*_//_ of the PEG@TPU/BNNS-es films. For example, the PEG@TPU/BNNS-es film shows an ultrahigh *κ*_//_ of 28.3 W m^−1^ K^−1^ at ~ 32 wt% BNNSs, which is ~ 16.5 times higher than that of PEG@TPU. Such a high *κ*_//_ is mainly attributed to the aligned and overlapping interconnected BNNS network along the in-plane direction of the nanocomposite films by electrostatic spraying. Furthermore, hot-pressing densifies the nanocomposite films, which is also beneficial to enhance the heat conduction. By contrast, the through-plane thermal conductivity (*κ*_┴_) is low (~ 0.9 W m^−1^ K^−1^), resulting in an obvious anisotropic heat conduction (Fig. S6). Figure 3e summarizes *κ*_//_ of the PEG@TPU/BNNS-es film and the PCCs reported previously [32, 33, 39–47]. The PEG@TPU/BNNS-es have prominently high *κ*_//_ in comparison with the reported PCCs containing comparable filler loading, indicating a great superiority of the aligned and overlapping interconnected BNNS network in thermal conductivity enhancement. Apart from exhibiting ultrahigh *κ*_//_, the PEG@TPU-BNNS-es films still have relatively high latent heat of > 101 J g^−1^, which provides a trade-off solution for two often paradoxical objective (i.e., high heat conduction and high heat storage), showing strong superiority in comparison with previously reported PCCs with polymeric supporting materials (Fig. 3f) [32, 33, 41–47].

### Dielectric and Mechanical Properties of the PCNs

A reliable electrical insulation performance is an important consideration for thermal management materials in power equipment and electronic devices. Compared with TPU, the incorporation of PEG results in a significant increase in electrical conductivity of PEG@TPU (Fig. S7a). As desired, however, the addition of 4.7 wt% BNNSs leads to an approximately one-order-of-magnitude decrease in electrical conductivity of PEG@TPU/BNNS, while the electrical conductivity is still higher than that of pure TPU. In the case of PEG@TPU/BNNS-es, a slightly decreased electrical conductivity is observed at low frequencies. This is because the aligned and interconnected BNNS network blocks the movement of charge carriers. The electrical conductivity of PEG@TPU/BNNS-es rises with increasing temperature, but it is still lower than 10^–10^ S m^−1^ at 60 °C (Fig. S7b), suggesting that PEG@TPU/BNNS-es remains electrical insulating above the phase transition temperature.

The electric breakdown of different films was analyzed by the Weibull distribution (Fig. S7c, d) according to *P*(*E*) = 1 − exp [− (*E* − *E*_th_)/*α*]^*β*^ [48], where *P*(*E*) is the cumulative probability of breakdown; *E* represents the experimental breakdown strength; *E*_th_ is the positional parameter, reflecting the threshold breakdown strength (*E*_th_ ≤ E); *α* is a scale parameter and *β* is a shape parameter for the data dispersion. Clearly, the PEG PEG@TPU/BNNS-es films show the highest *E*_th_ values among TPU, PEG@TPU, PEG@TPU/BNNS and PEG@TPU/BNNS-es films. This is because the aligned and overlapping interconnected BNNSs can form a robust scaffold that effectively hampers the onset of the electromechanical failure [36].

**Fig. 4:**
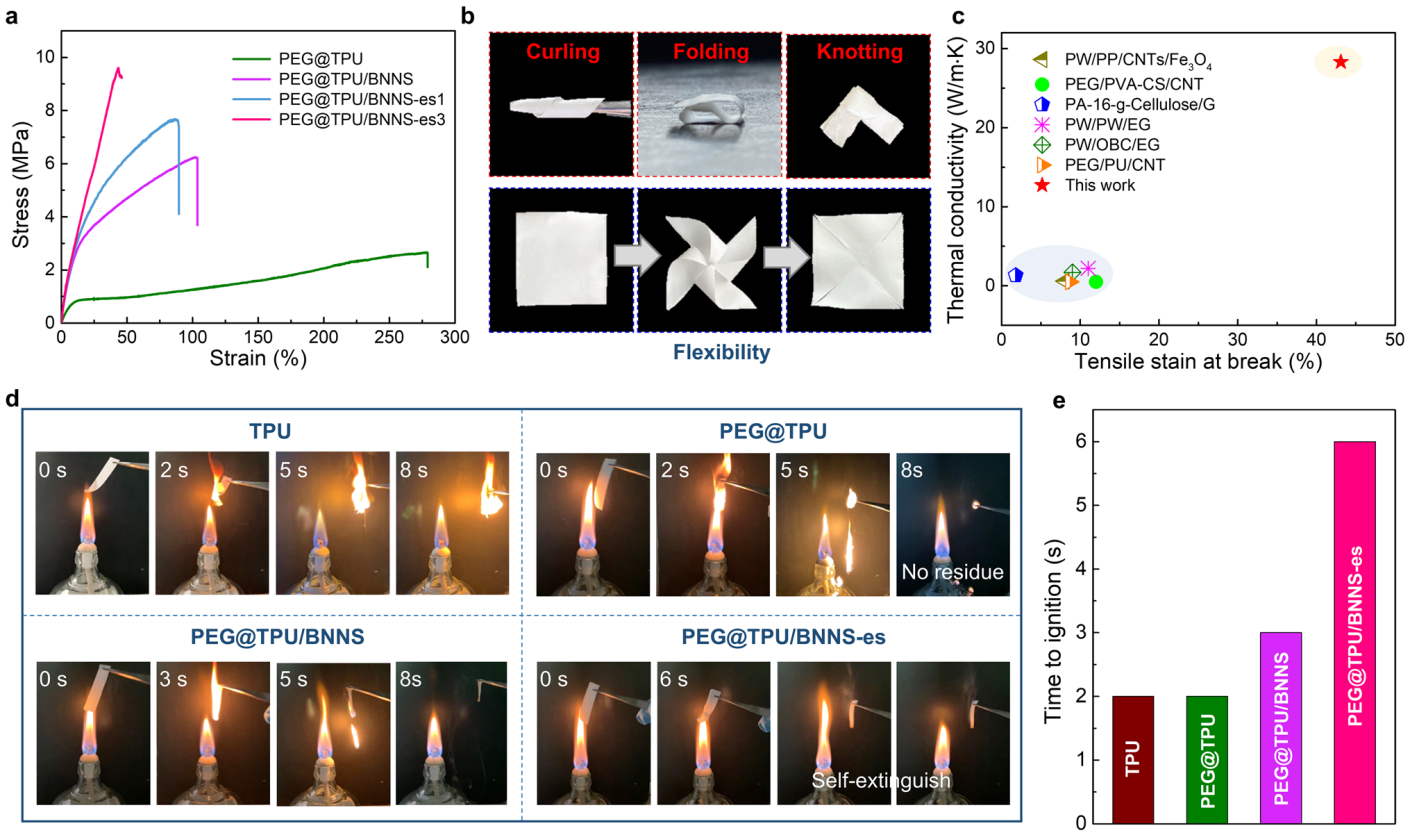
Mechanical and fire-retardant properties of various materials. **a** Stress–strain curves of the core–sheath PCCs with different BNNS loading. **b** Optical photographs of the PEG@TPU/BNNS-es film under curling, folding and knotting conditions (top), and before and after shape recovery (bottom). **c** Thermal conductivity versus elongation at break of PEG@TPU/BNNS-es films and previously reported flexible PCNs [31, 33, 41, 47, 49, 50]. **d** Optical photographs showing the burning of various material by an alcohol lamp. **e** Ignition delay time of TPU and different PCNs

The introduction of TPU endows the PCNs with excellent flexibility. The PEG@TPU exhibits no breaks until the strain is up to ~ 280% at an imposed stress of 2.65 MPa. The elongation at break of the composite fiber films decreases with increase in BNNS fraction (Figs. 4a and S8), while the tensile strength increases as BNNS increases. Nonetheless, the PEG@TPU/BNNS-es with 32 wt% BNNS still exhibit a high strain of 45% at a stress of 9.2 MPa, demonstrating both excellent flexibility and strength of the PEG@TPU/BNNS-es films. In this case, the PEG@TPU/BNNS-es film can be curled, folded, and knotted (Fig. 4b). It can even be bent and fixed to various complex shapes, such as a small windmill, and the shape deformation of the film can be almost restored to the original state after removing the pins. Figure 4c summarizes the thermal conductivity and elongation at break of flexible PCCs reported in previous studied [31, 33, 41, 47, 49, 50]. Apparently, our PEG@TPU/BNNS-es exhibits the highest flexibility and largest thermal conductivity, confirming the superiority of the aligned and interconnected BNNS network to enhance both thermal conductivity and flexibility of these films.

### Flame Retardant Property

Most polymeric PCMs are flammable, which can easily cause fire risk in practical applications. As shown in Fig. 4d, e**,** TPU and PEG@TUP films can be immediately ignited (within 2 s) by an alcohol lamp and combust quickly. In addition, we can see a significant volume contraction in pure TPU film during combustion, and it burns fiercely even after removing the flame. Similarly, the PEG@TPU film also shows a violent combustion accompanied by melting dripping. As a result, it burns out completely without residue within only 8 s. In the case of PEG@TPU/BNNS, although the film can be ignited and combusts accompanied by melting dripping, it can stop further combustion after the burning droplets fall off. The PEG@TPU/BNNS-es film, however, can hardly be ignited by the flame within 6 s and it self-extinguishes immediately upon the flame is removed, illustrating excellent fire retardancy in the flame. The longer ignition time of PEG@TPU/BNNS-es is attributed to the integration of the BNNSs network, which effectively blocks the external heat attacking the interior of the PCNs.

### Applications of the PCN Films in 5G Base Station and Thermoelectric Power Generation

5G mobile communication has merits of faster transmission rates, lower transmission delays and higher capacity when compared with previous generations. It is the emerging and promising communication infrastructure to address the growing traffic demands of the next-generation mobiles and Internet of Things [51]. However, with the significant growth in energy consumption of 5G base stations, existing heat dissipation technologies can hardly fulfill the operation requirements of 5G hardware systems. In fact, a high operation temperature may cause automatic under-clocking of the chips to ensure the safety of the base station, which can inevitably reduce the transmission rate and exacerbate the transmission delays [37]. Therefore, it is of great importance to reduce the operation temperature of the chips to attain higher operation efficiency of 5G base stations. The thermal interface material (TIM) between the chip and the heat sink is the key component to improve the thermal dissipation [52].

**Fig. 5:**
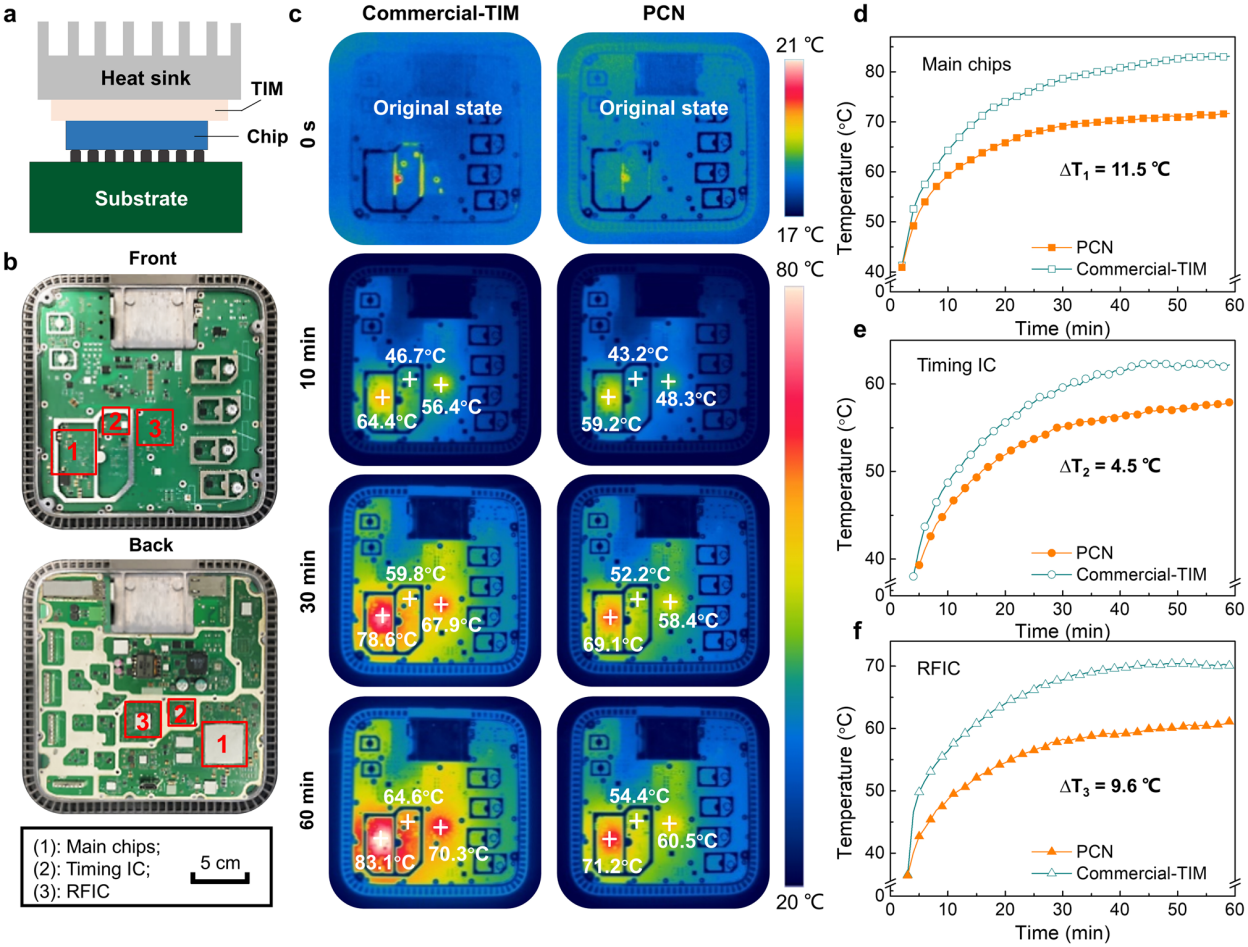
Thermal management application of PEG@TPU/BNNS-es (PCN) film in a 5G base station. **a** Schematic diagram showing a chip integrated with a TIM in a 5G base station. **b** Optical photographs showing the front side (top) and the back side (bottom) of the 5G base station (without cover), and the regions identified by red squares as regions 1–3, corresponding to the main chips, timing IC and RFIC, respectively. **c** Infrared thermal images of the 5G base station integrated with commercial and PCN TIMs. Surface temperature versus time in **d** region 1, **e** region 2 and **f** region 3 in the 5G base station integrated with commercial TIM and PCN

Thermal dissipation of the PEG@TPU/BNNS-es film as a TIM between the chips and the heat sink was evaluated in a 5G base station (Fig. 5a). Also, commercial TIM (i.e., thermally conductive silicone grease with a through-plane thermal conductivity of ~ 4.5 W m^−1^ K^−1^) was used as a control. This 5G base station contains three kinds of important chips, including main chips, timing chip and radio frequency (RF) chip. The corresponding integrated circuits (IC) are roughly distributed in the regions 1–3 indicated by red squares in Fig. 5b, respectively. The 5G base station began working at room temperature, then the surface temperature of the front side (Fig. 5b) was recorded by an infrared camera during a 60-min operation (Figs. 5c–f, S9a–e). Clearly, the base station integrated with the PEG@TPU/BNNS-es film shows slower temperature rise and lower steady-state temperature when compared with the counterpart using commercial TIM. Specifically, the steady-state temperature differences (Δ*T*) in the regions 1–3 where the main chips, timing IC and RF IC are located are 11.5, 4.5 and 9.6 °C, respectively.

The infrared thermographs mainly capture the surface temperature of the front side of the 5G base station. To accurately reflect the actual thermal performance of the chips, we also recorded the chip temperature through the system program (Fig. S9f) after the 60-min operation. We can observe that the internal temperatures of the chips are much higher than the corresponding surface temperatures recorded by the thermographs. Further, compared with the commercial TIM, the PEG@TPU/BNNS-es film results in about 18.5, 11.8 and 18 °C temperature drops of main chips, timing IC and RFIC, respectively. This result suggests that the PEG@TPU/BNNS-es film has much more superior heat dissipation capability than the results measured by the infrared thermographs. The prominent heat dissipation capability of the PEG@TPU/BNNS-es films could be attributed to the ultrahigh in-plane thermal conductivity and excellent flexibility. The former effectively addresses the ‘hot spot’ formed by the generated heat of the chips, while the latter enables a tighter fit between the TIM and the contact surfaces, resulting in reduced contact thermal resistance.

**Fig. 6:**
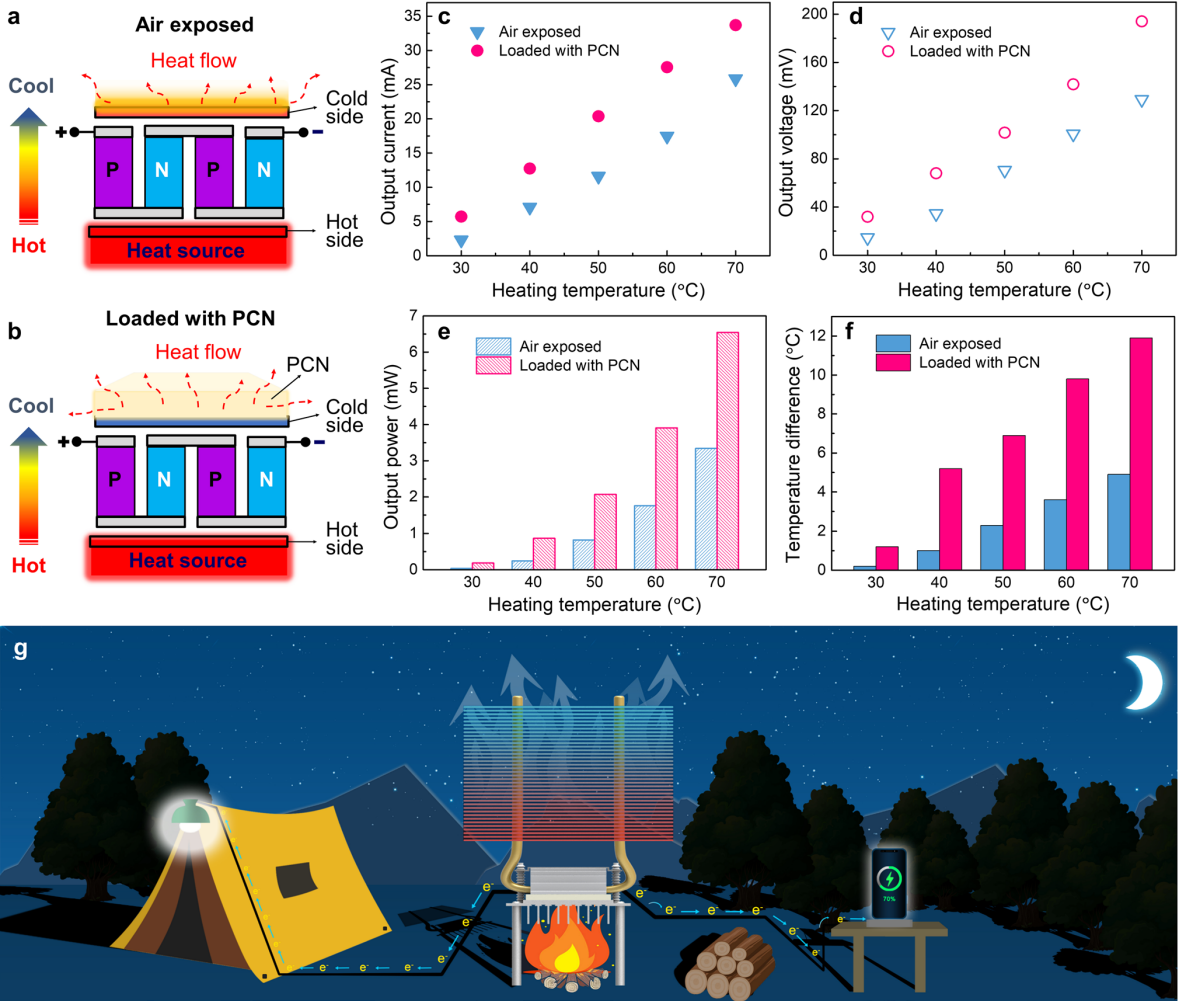
Thermal management application of PEG@TPU/BNNS-es film (with 32 wt% BNNSs content) in thermoelectric generator (TEG). **a** Schematic mechanism of TEG exposed to air (top) and **b** loaded with PEG@TPU/BNNS-es (PCN) (bottom). **c–e** Output current, output voltage and output power of TEGs with/without PCC at different heating temperature, respectively. **f** Temperature difference between the hot and cold sides versus heating temperature of TEGs. **g** Conception diagram of potential application of TEGs integrated with the PEG@TPU/BNNS-es film for outdoor activities

Thermoelectric generators (TEGs) can directly convert heat energy to electrical energy based on the Seebeck effect, which makes it available as an emergency power source [53]. The power generation efficiency depends on the temperature difference between the cold and hot surfaces of a TEG [54]. PCMs are capable of absorbing a large amount of heat from the cold side and thus it can enhance the power generation efficiency of TEGs by increasing the temperature difference [38]. The PEG@TPU/BNNS-es, as a heat sink, was used on the cold side of a TEG to enhance the power generation (Fig. 6a, b). Notably, the TEG loaded with PEG@TPU/BNNS-es exhibits higher output current and output voltage in comparison with the device exposed to air (Fig. 6c, d). Specifically, under the heating temperature of 70 °C, the output current and output voltage of the TEG loaded with PEG@TPU/BNNS-es are 34 mA and 194 mV, which are, respectively, 30.5% and 50.2% higher than those of the unloaded device. Since the heat storage capability is related to the volume of the PCMs, the cooling efficiency can increase further by increasing the size/volume of the PCNs. In addition, the higher output power of the TEG also suggests that loading of PEG@TPU/BNNS-es enhances the energy conversion efficiency of the device (Fig. 6e). Under 70 °C heating temperature, the loaded TEG shows a 100% output power enhancement than that of the device exposed to air. The enhanced output properties of the TEG loaded with PEG@TPU/BNNS-es benefit from the much larger temperature differences between the cold and the hot sides (Fig. 6f)**.** These results prove the excellent thermal management performance of the PEG@TPU/BNNS-es in thermoelectric power generation.

The ultrahigh-thermal-conductivity and flexible PEG@TPU/BNNS-es film was further used as a TIM in a high-power TEG (Fig. S10a). This TEG does not only light up an LED light bulb, but also charge the cellphone (Fig. S10b, c, Video S1). Here, the TEG integrated with PEG@TPU/BNNS-es can provide emergency power supply for outdoor activities, such as a camping trip and a scientific expedition. Particularly, the TEG integrated with PEG@TPU/BNNS-es, which uses burning firewood as a heating source, can work well at night or on overcast days when solar power generation facilities fail to work (Fig. 6g). Therefore, the flexible PEG@TPU/BNNS-es with ultrahigh in-plane thermal conductivity as well as good fire retardancy have tremendous applications in thermoelectric generators, providing emergency power for lighting and electronic devices.

## Conclusions

In this work, core–sheath structured PCNs (e.g., PEG@TPU/BNNS-es) with aligned and overlapping interconnected BNNS networks were designed and fabricated by coaxial electrospinning, electrostatic spraying and hot-pressing. The resultant PCNs simultaneously display an ultrahigh in-plane thermal conductivity (28.3 W m^−1^ K^−1^), a high strain to break (45%), and a high phase change enthalpy (101 J g^−1^). In addition, these PCN films have good flame-retardancy and retain electrical insulation even at the phase transition temperature. The core–sheath PCNs significantly enhance the heat dissipation of 5G base station chips, avoiding the automatic under-clocking of the chips due to overheating. Moreover, they can largely increase the temperature differences of the TEGs, resulting in significantly enhanced output properties. These results demonstrate that the PCNs have broad application prospects in thermal management of high-power-density electric equipment and emerging electronic devices.

### Supplementary Information

Below is the link to the electronic supplementary material.

Supplementary file1 (PDF 1491 KB)

Supplementary file2 (mp4 1491 kb)

## References

[1] Sun B, Huang X (2021). Seeking advanced thermal management for stretchable electronics. npj Flex. Electron..

[2] Yan Q, Alam FE, Gao J, Dai W, Tan X (2021). Soft and self-adhesive thermal interface materials based on vertically aligned, covalently bonded graphene nanowalls for efficient microelectronic cooling. Adv. Funt. Mater..

[3] Gong J, Tan X, Yuan Q, Liu Z, Ying J (2021). A spiral graphene framework containing highly ordered graphene microtubes for polymer composites with superior through-plane thermal conductivity. Chin. J. Chem..

[4] Lin Y, Kang Q, Wei H, Bao H, Jiang P (2021). Spider web-inspired graphene skeleton-based high thermal conductivity phase change nanocomposites for battery thermal management. Nano Micro Lett..

[5] Ling Z, Zhang Z, Shi G, Fang X, Wang L (2014). Review on thermal management systems using phase change materials for electronic components, li-ion batteries and photovoltaic modules. Renew. Sustain. Energy Rev..

[6] Wu S, Li T, Tong Z, Chao J, Zhai T (2019). High-performance thermally conductive phase change composites by large-size oriented graphite sheets for scalable thermal energy harvesting. Adv. Mater..

[7] Yan Q, Dai W, Gao J, Tan X, Lv L (2021). Ultrahigh-aspect-ratio boron nitride nanosheets leading to superhigh in-plane thermal conductivity of foldable heat spreader. ACS Nano.

[8] Lin Y, Chen J, Dong S, Wu G, Jiang P (2021). Wet-resilient graphene aerogel for thermal conductivity enhancement in polymer nanocomposites. J. Mater. Sci. Technol..

[9] Min P, Liu J, Li X, An F, Liu P (2018). Thermally conductive phase change composites featuring anisotropic graphene aerogels for real-time and fast-charging solar-thermal energy conversion. Adv. Funt. Mater..

[10] Yu H, Guo P, Qin M, Han G, Chen L (2022). Highly thermally conductive polymer composite enhanced by two-level adjustable boron nitride network with leaf venation structure. Compos. Sci. Technol..

[11] Wang B, Li G, Xu L, Liao J, Zhang X (2020). Nanoporous boron nitride aerogel film and its smart composite with phase change materials. ACS Nano.

[12] Li Y, Li Y, Huang X, Zheng H, Lu G (2020). Graphene-coo/peg composite phase change materials with enhanced solar-to-thermal energy conversion and storage capacity. Compos. Sci. Technol..

[13] Zhu Y, Shen Z, Li Y, Chai B, Chen J (2022). High conduction band inorganic layers for distinct enhancement of electrical energy storage in polymer nanocomposites. Nano Micro Lett..

[14] Huang L, Xiao G, Wang Y, Li H, Zhou Y (2022). Self-exfoliation of flake graphite for bioinspired compositing with aramid nanofiber toward integration of mechanical and thermoconductive properties. Nano Micro Lett..

[15] Yu H, Feng Y, Chen C, Zhang H, Peng L (2022). Highly thermally conductive adhesion elastomer enhanced by vertically aligned folded graphene. Adv. Sci..

[16] Chen J, Shen Z, Kang Q, Qian X, Li S (2022). Chemical adsorption on 2D dielectric nanosheets for matrix free nanocomposites with ultrahigh electrical energy storage. Sci. Bull..

[17] Yuan K, Shi J, Aftab W, Qin M, Usman A (2019). Engineering the thermal conductivity of functional phase-change materials for heat energy conversion, storage, and utilization. Adv. Funt. Mater..

[18] Jebasingh BE, Arasu AV (2020). A comprehensive review on latent heat and thermal conductivity of nanoparticle dispersed phase change material for low-temperature applications. Energy Storage Mater..

[19] Guo C, He L, Yao Y, Lin W, Zhang Y (2022). Bifunctional liquid metals allow electrical insulating phase change materials to dual-mode thermal manage the li-ion batteries. Nano Micro Lett..

[20] Yang J, Zhou YC, Yang LY, Feng CP, Bai L (2022). Exploring next-generation functional organic phase change composites. Adv. Funt. Mater..

[21] Zhang X, Liu H, Huang Z, Yin Z, Wen R (2016). Preparation and characterization of the properties of polyethylene glycol@ Si_3_N_4_ nanowires as phase-change materials. Chem. Eng. J..

[22] Kou Y, Sun K, Luo J, Zhou F, Huang H (2021). An intrinsically flexible phase change film for wearable thermal managements. Energy Storage Mater..

[23] Lu Y, Xiao X, Fu J, Huan C, Qi S (2019). Novel smart textile with phase change materials encapsulated core-sheath structure fabricated by coaxial electrospinning. Chem. Eng. J..

[24] Wang J, Huang X, Gao H, Li A, Wang C (2018). Construction of CNT@Cr-MIL-101-NH_2_ hybrid composite for shape-stabilized phase change materials with enhanced thermal conductivity. Chem. Eng. J..

[25] Cheng P, Chen X, Gao H, Zhang X, Tang Z (2021). Different dimensional nanoadditives for thermal conductivity enhancement of phase change materials: Fundamentals and applications. Nano Energy.

[26] Aftab W, Huang X, Wu W, Liang Z, Mahmood A (2018). Nanoconfined phase change materials for thermal energy applications. Energy Environ. Sci..

[27] Gao D-C, Sun Y, Fong AML, Gu X (2022). Mineral-based form-stable phase change materials for thermal energy storage: a state-of-the art review. Energy Storage Mater..

[28] Yu D-H, He Z-Z (2019). Shape-remodeled macrocapsule of phase change materials for thermal energy storage and thermal management. Appl. Energy.

[29] Atinafu DG, Yun BY, Yang S, Yuk H, Wi S (2021). Structurally advanced hybrid support composite phase change materials: architectural synergy. Energy Storage Mater..

[30] Zhao X, Zou D, Wang S (2022). Flexible phase change materials: Preparation, properties and application. Chem. Eng. J..

[31] Qian Y, Han N, Zhang Z, Cao R, Tan L (2019). Enhanced thermal-to-flexible phase change materials based on cellulose/modified graphene composites for thermal management of solar energy. ACS Appl. Mater. Interfaces.

[32] He Y, Li H, Luo F, Jin Y, Huang B (2021). Bio-based flexible phase change composite film with high thermal conductivity for thermal energy storage. Compos. Part A Appl. Sci. Manuf..

[33] Li W-W, Cheng W-L, Xie B, Liu N, Zhang L-S (2017). Thermal sensitive flexible phase change materials with high thermal conductivity for thermal energy storage. Energy Convers. Manage..

[34] Gong S, Li X, Sheng M, Liu S, Zheng Y (2021). High thermal conductivity and mechanical strength phase change composite with double supporting skeletons for industrial waste heat recovery. ACS Appl. Mater. Interfaces.

[35] Lu Y, Xiao X, Zhan Y, Huan C, Qi S (2018). Core-sheath paraffin-wax-loaded nanofibers by electrospinning for heat storage. ACS Appl. Mater. Interfaces.

[36] Chen J, Huang X, Sun B, Jiang P (2019). Highly thermally conductive yet electrically insulating polymer/boron nitride nanosheets nanocomposite films for improved thermal management capability. ACS Nano.

[37] Chen Y, Zhang H, Chen J, Guo Y, Jiang P (2022). Thermally conductive but electrically insulating polybenzazole nanofiber/boron nitride nanosheets nanocomposite paper for heat dissipation of 5G base stations and transformers. ACS Nano.

[38] Wang T, Lin Y, Li P, Jiang P, Zhang C (2022). Unidirectional thermal conduction in electrically insulating phase change composites for superior power output of thermoelectric generators. Compos. Sci. Technol..

[39] Chang C, Nie X, Li X, Tao P, Fu B (2020). Bioinspired roll-to-roll solar-thermal energy harvesting within form-stable flexible composite phase change materials. J. Mater. Chem. A.

[40] Huang Q, Deng J, Li X, Zhang G, Xu F (2020). Experimental investigation on thermally induced aluminum nitride based flexible composite phase change material for battery thermal management. J. Energy Storage.

[41] Cai Z, Liu J, Zhou Y, Dai L, Wang H (2021). Flexible phase change materials with enhanced tensile strength, thermal conductivity and photo-thermal performance. Sol. Energy Mater. Sol. Cells.

[42] Zhang W, Zhang X, Xu Y, Xu Y, Qiao J (2021). Flexible polyethylene glycol/polyvinylpyrrolidone composite phase change fibres: preparation, characterization, and thermal conductivity enhancement. Polymer.

[43] Wu W, Wu W, Wang S (2019). Form-stable and thermally induced flexible composite phase change material for thermal energy storage and thermal management applications. Appl. Energy.

[44] Luo D, Wei F, Shao H, Xiang L, Yang J (2018). Shape stabilization, thermal energy storage behavior and thermal conductivity enhancement of flexible paraffin/mwcnts/pp hollow fiber membrane composite phase change materials. J. Mater. Sci..

[45] Huang Y-H, Cheng W-L, Zhao R (2019). Thermal management of li-ion battery pack with the application of flexible form-stable composite phase change materials. Energy Convers. Manage..

[46] Huang Q, Li X, Zhang G, Deng J, Wang C (2021). Thermal management of lithium-ion battery pack through the application of flexible form-stable composite phase change materials. Appl. Therm. Eng..

[47] Shi J, Aftab W, Liang Z, Yuan K, Maqbool M (2020). Tuning the flexibility and thermal storage capacity of solid–solid phase change materials towards wearable applications. J. Mater. Chem. A.

[48] Nohut S (2021). Three-parameter (3P) Weibull distribution for characterization of strength of ceramics showing R-curve behavior. Ceram. Int..

[49] Li X, Sheng M, Gong S, Wu H, Chen X (2022). Flexible and multifunctional phase change composites featuring high-efficiency electromagnetic interference shielding and thermal management for use in electronic devices. Chem. Eng. J..

[50] Cheng P, Gao H, Chen X, Chen Y, Han M (2020). Flexible monolithic phase change material based on carbon nanotubes/chitosan/poly(vinyl alcohol). Chem. Eng. J..

[51] Israr A, Yang Q, Israr A (2022). Power consumption analysis of access network in 5G mobile communication infrastructures—an analytical quantification model. Pervasive Mob. Comput..

[52] Liu P, Li X, Min P, Chang X, Shu C (2020). 3D lamellar-structured graphene aerogels for thermal interface composites with high through-plane thermal conductivity and fracture toughness. Nano Micro Lett..

[53] Tian Y, Liu A, Wang J, Zhou Y, Bao C (2021). Optimized output electricity of thermoelectric generators by matching phase change material and thermoelectric material for intermittent heat sources. Energy.

[54] Wang J, Liu D, Li Q, Chen C, Chen Z (2019). Lightweight, superelastic yet thermoconductive boron nitride nanocomposite aerogel for thermal energy regulation. ACS Nano.

