# Supplementary material for: Flexible, Highly Thermally Conductive and Electrically Insulating Phase Change Materials for Advanced Thermal Management of 5G Base Stations and Thermoelectric Generators
Source: Nanomicro Lett. 2023 Jan 9;15:31. doi: 10.1007/s40820-022-01003-3 (PMC9829950; doi:10.1007/s40820-022-01003-3)
Supplement: Supplementary file 1 — Supplementary file1 (PDF 1491 KB) [file 40820_2022_1003_MOESM1_ESM.pdf]

Supporting Information for

Flexible, Highly Thermally Conductive and Electrically Insulating Phase Change Materials for Advanced Thermal Management of 5G Base Stations and Thermoelectric Generators

Ying Lin[1], Qi Kang[1], Yijie Liu[1], Yingke Zhu[1], Pingkai Jiang[1], Yiu-Wing Mai[2], Xingyi Huang[1, *]

[1]Shanghai Key Laboratory of Electrical Insulation and Thermal Ageing, Department of Polymer Science and Engineering, the State Key Laboratory of Metal Matrix Composites, Shanghai Jiao Tong University, Shanghai 200240, P. R. China

[2]Centre for Advanced Materials Technology (CAMT), School of Aerospace, Mechanical and Mechatronic Engineering J07, The University of Sydney, Sydney, NSW 2006, Australia

*Corresponding author. E-mail: xyhuang@sjtu.edu.cn (Xingyi Huang)

Supplementary Figures

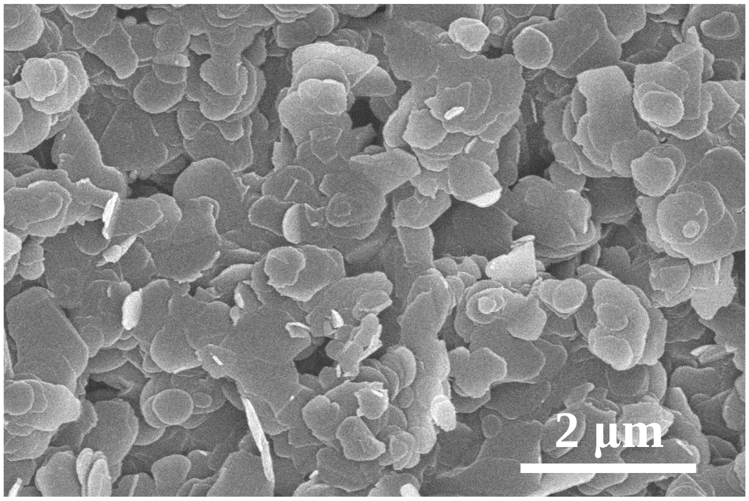

Fig. S1 SEM image of BNNSs

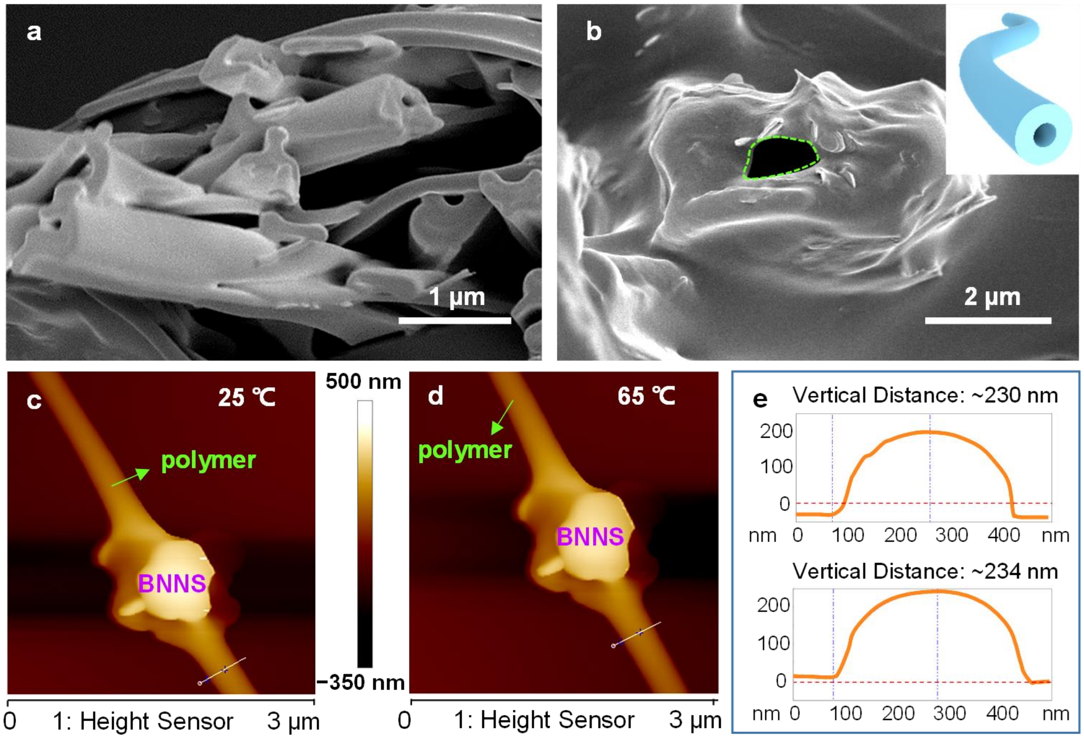

Fig. S2 a-b SEM images of the core-sheath structured phase change composite fibers treated with 90 °C water washing for 24 h. **c-d** AFM images of the PEG@TPU/BNNS fiber tested at 25 and 65 °C, respectively. **e** Corresponding AFM data of the PEG@TPU/BNNS fiber at 25 (top) and 65 °C (bottom)

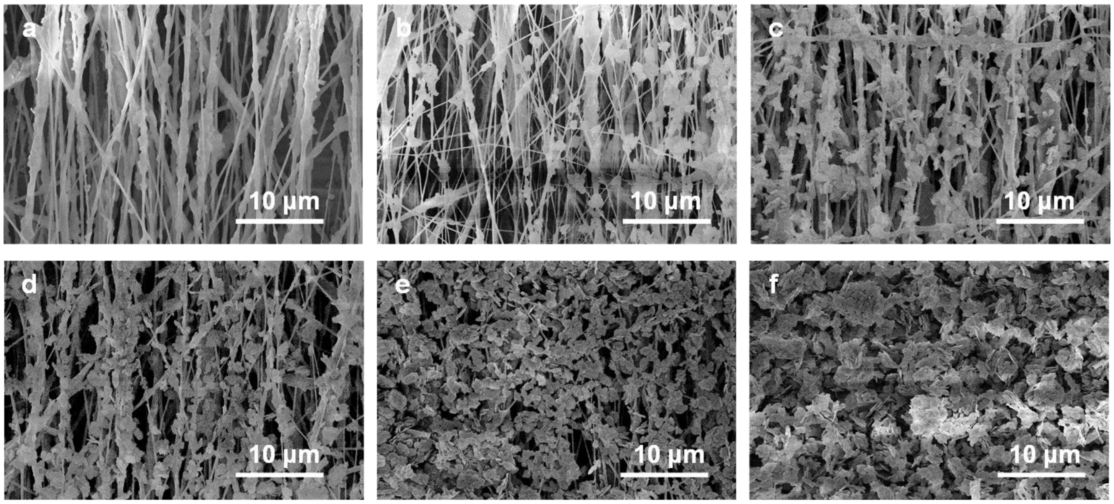

Fig. S3 SEM images of the phase change nanocomposite fibers with BNNS spraying time of **a** 0, **b** 20, **c** 40, **d** 60, **e** 80 and **f** 100 min

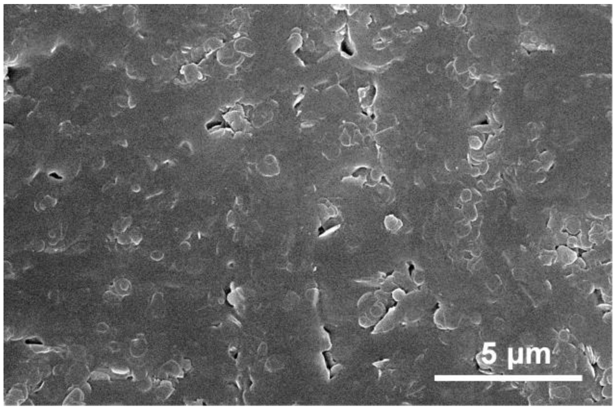

Fig. S4 SEM image of the surface of PEG@TPU/BNNS-es nanocomposites (after hot pressing)

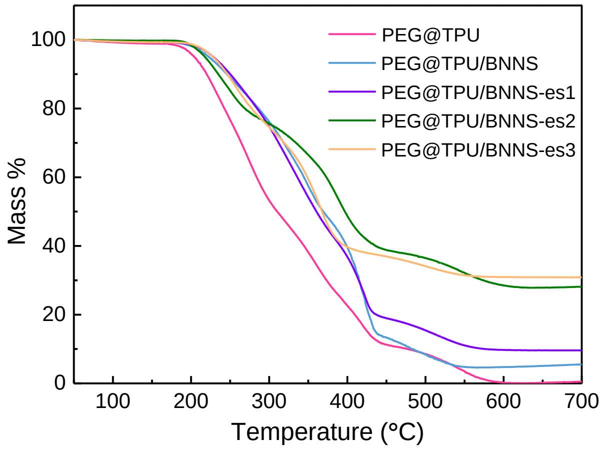

Fig. S5 Thermogravimetric curves of the phase change nanocomposites with different BNNS content

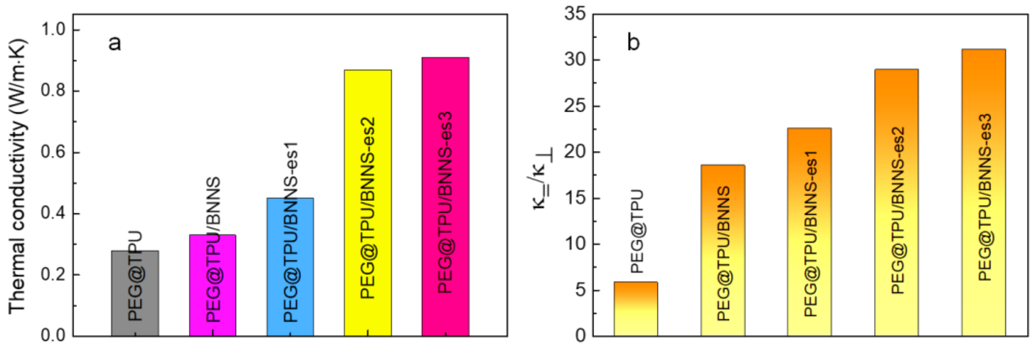

Fig. S6 a Through-plane thermal conductivities (κ⊥) of the phase change nanocomposites with different BNNS content. **b** The thermal conductivity anisotropy ($\kappa_{//}$ / κ⊥) of the phase change nanocomposites

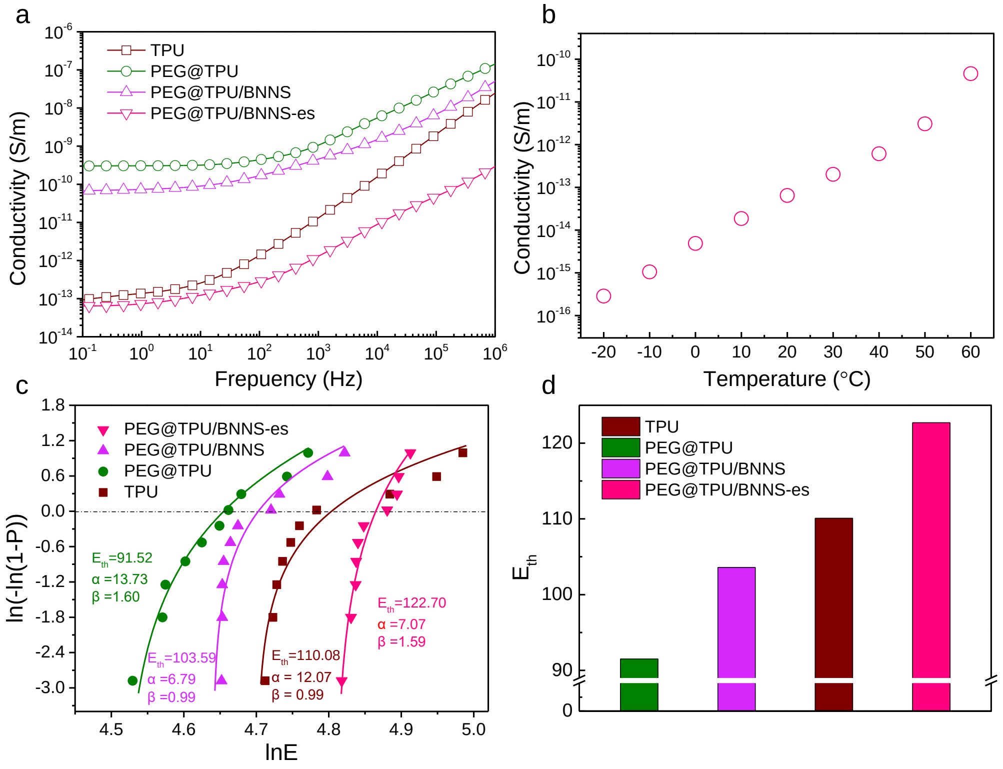

Fig. S7 a Frequency-dependent electrical conductivities at room temperature of electrospun TPU film and the phase change nanocomposites. **b** The electrical conductivities of the PEG@TPU/BNNS-es at various temperatures (-20, -10, 0, 10, 20, 30, 40, 50 and 60 °C) of the phase change nanocomposite films. **c** The 3-parameter Weibull plots of breakdown strength of different polymer and phase change nanocomposite electrospun films, the value of goodness of fit (R^2) for each of these 4 curves of TPU, PEG@TPU, PEG@TPU/BNNS and PEG@TPU/BNNS-es are 0.9765, 0.9947, 0.9645 and 0.9709, respectively. **d** The threshold breakdown strength (E_{th}, $E_{th} \leq$ E) of different polymer and phase change nanocomposite electrospun films

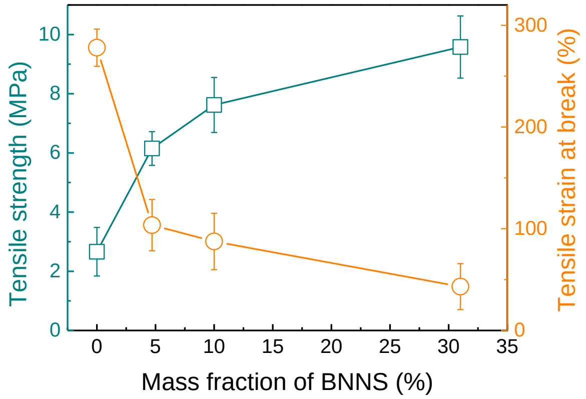

Fig. S8 The tensile strength and elongation at break of the phase change nanocomposites with different BNNS loading

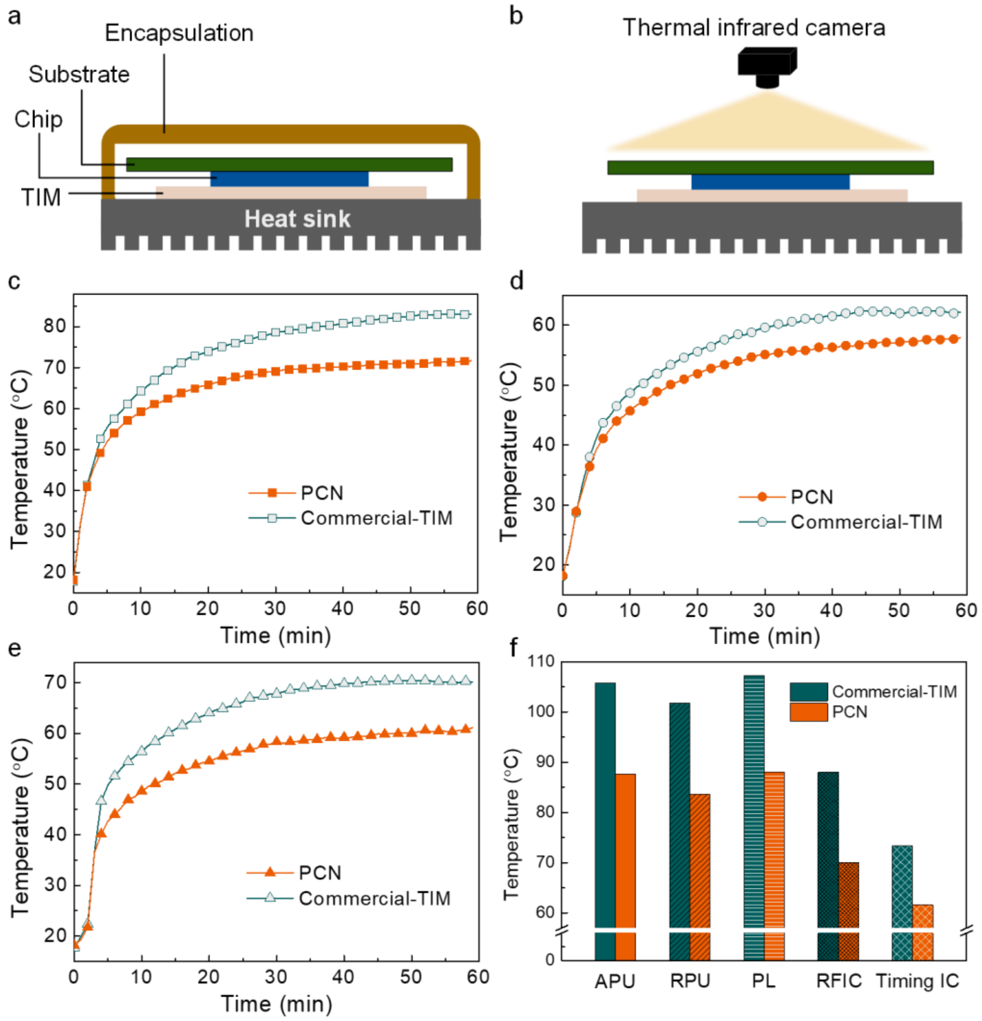

Fig. S9 a Schematic diagram of the 5G base station. **b** Schematic diagram of using a thermal infrared camera to record the surface temperature of the front side of the base station during operation. The encapsulation (shell) of the base station was removed during the test. **c-e** The surface temperature variation curves of the regions 1-3 (see **Figure 5**a,b) in the 5G base station during the operation process, respectively. **f** The maximum temperature values of the chips obtained from the system program during operation. The main chips contain accelerated processing unit (APU), radio processing unit (RPU) and PL

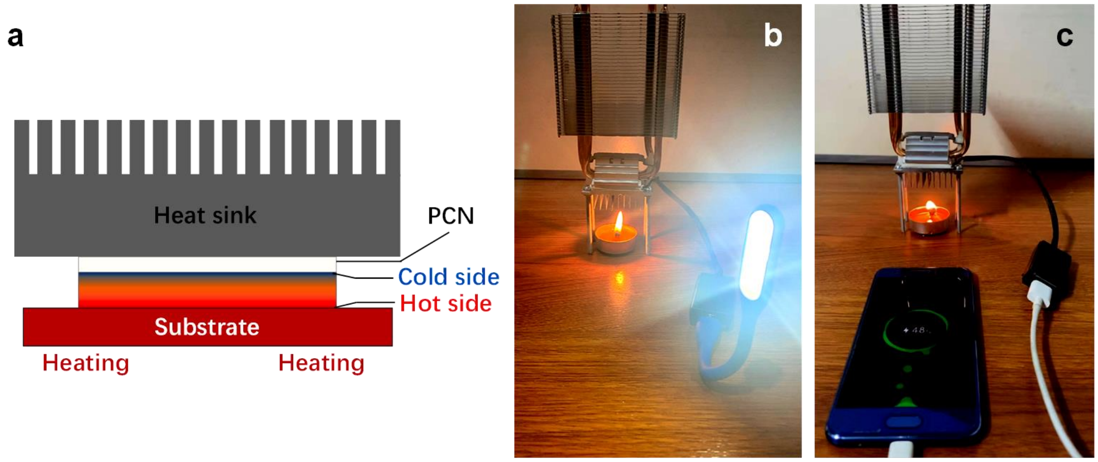

Fig. S10 a Schematic diagram showing a thermoelectric generator (TEG) integrated with PCNs (the PCN can be used not only as a TIM, but also a heat sink). **b-c** Optical photographs of the TEG integrated with PCNs providing power for lighting and a cellphone

Table S1 DSC heating and cooling characteristics of polymer and the PCNs in the temperature range of 20-90 °C

Sample	T_m (°C)	ΔH_m (J/g)	T_s (°C)	ΔH_s (J/g)
**TPU**	-	-	-	-
**PEG@TPU**	61.0	156.5	36.8	154.1
**PEG@TPU/BNNS**	60.2	132.5	37.9	130.3
**PEG@TPU/BNNS-es**	61.4	102.9	34.9	101.2
**PEG**	60.7	173.8	38.6	170.2
